# Cubitus varus after pediatric lateral condylar fracture: true or pseudo?

**DOI:** 10.1186/s12891-023-06604-6

**Published:** 2023-06-13

**Authors:** Kyungil Kim, Chiyoung Yoon, Han Yong Lee

**Affiliations:** 1 Department of Orthopedic Surgery, Wonkwang University Sanbon Hospital, Gunpo, Republic of Korea; 2 Department of Orthopedic Surgery, St. Vincent’s Hospital, College of Medicine, The Catholic University of Korea, Seoul, Republic of Korea

**Keywords:** Pediatric lateral condylar fracture, cubitus varus, Pseudo-cubitus varus, Lateral condylar overgrowth, Interepicondylar width

## Abstract

**Purpose:**

Common complications of lateral condylar fractures are lateral condylar overgrowth, lateral bony spur and cubitus varus. Lateral condylar overgrowth or lateral bony spur may appear as cubitus varus on gross examination. Such gross cubitus varus without actual angulation is pseudo-cubitus varus, while a difference of more than 5° in varus angulation on X-ray is true cubitus varus. This study aimed to compare true and pseudo-cubitus varus.

**Methods:**

One hundred ninety-two children treated for unilateral lateral condylar fracture with a follow-up period of over six months were included. The Baumann angle, humerus-elbow-wrist angle and interepicondylar width of both side were compared. More than 5° in varus angulation on X-ray was considered cubitus varus. Increase in interepicondylar width was considered lateral condylar overgrowth or a lateral bony spur. The risk factors that could predict the development of a true cubitus varus were analyzed.

**Results:**

True cubitus varus was 32.8%, measured by Baumann angle and 29.2%, measured by humerus-elbow-wrist angle. A total of 94.8% of patients showed an increased interepicondylar width. The predicted cut-off value for 5° varus angulation on the Baumann angle was a 3.675 mm increase in interepicondylar width by ROC curve analysis. The risk of cubitus varus in stage 3, 4, and 5 fractures according to Song’s classification was 2.88 times higher than that in stage 1 and 2 fractures on multivariable logistic regression analysis.

**Conclusion:**

Pseudo-cubitus varus is more prevalent than true cubitus varus. A 3.7 mm increase in interepicondylar width could simply predict true cubitus varus. The risk of cubitus varus increased in Song’s classification stages 3, 4, and 5.

## Introduction

**Fig. 1:**
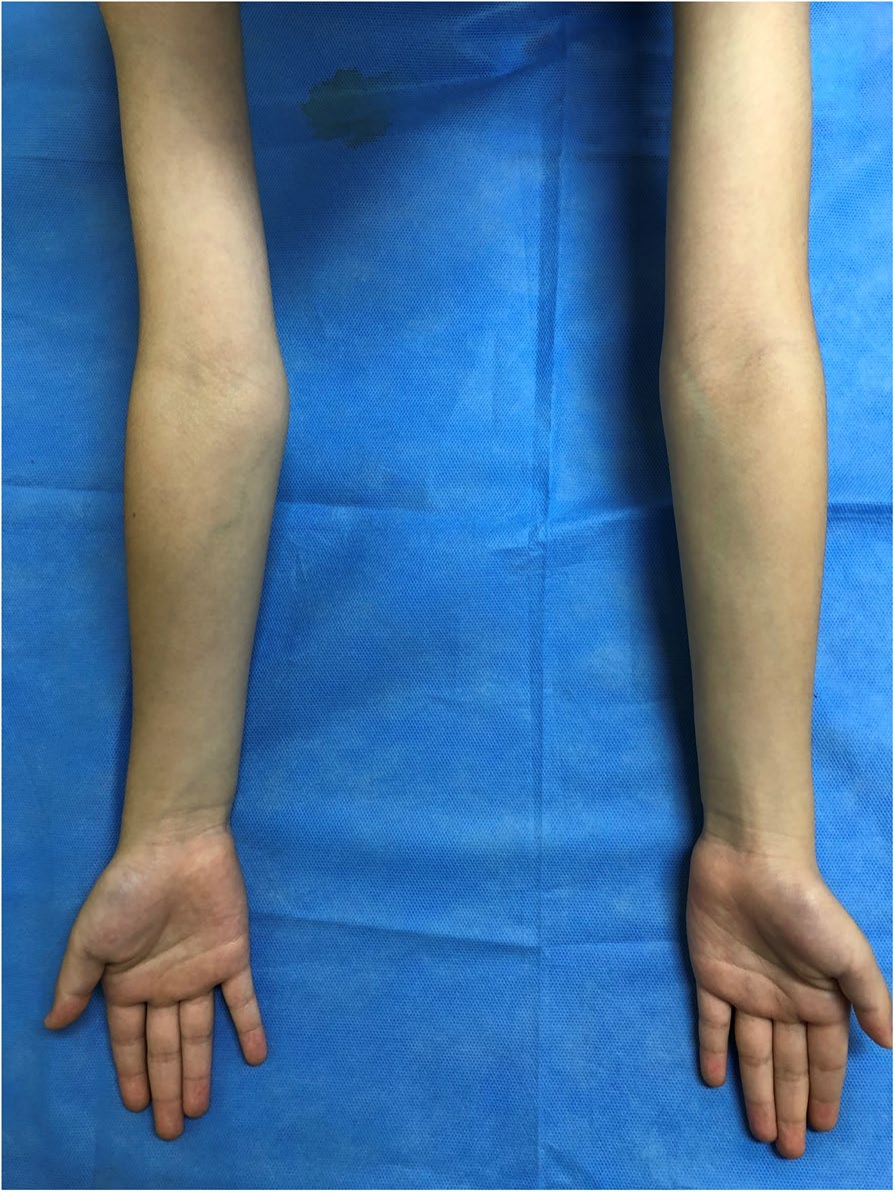
Left elbow pseudo-cubitus varus after pediatric lateral condylar fracture of the distal humerus. The right elbow is the unaffected side. Cubitus varus appearance of the left elbow on gross examination, with lateral condylar overgrowth or a lateral bony spur but without more than 5° varus angulation compared to the right side

Pediatric lateral condylar fractures of the distal humerus are the second most common fracture around the elbow [1–3]. The clinical results are usually acceptable with adequate reduction and solid fixation [4–6]. However, most patients who have healed from this injury have quite identifiable X-rays, regardless of Song’s classification fracture stage [4] or treatment method. Some complications occur after lateral condyle fracture, such as cubitus varus, cubitus valgus, non-union, avascular necrosis, pre-mature epiphyseal fusion, lateral condyle overgrowth, stiffness, and fishtail deformity [5, 7–9]. Among them, lateral overgrowth and cubitus varus are the most commonly reported after lateral condyle fracture [10]. Lateral condylar overgrowth or lateral bony spur has a prevalence of up to 70% [10], while that of cubitus varus is reportedly up to 40% [11]. Lateral condylar overgrowth or lateral bony spur may appear grossly in cubitus varus (Fig. 1). This gross cubitus varus without actual angulation is pseudo-cubitus varus, whereas greater than 5° varus angulation on X-ray is true cubitus varus [12]. These two common complications may look similar, but there is a significant difference. This study aimed to: (1) compare the incidence of pseudo-cubitus varus and true cubitus varus by the measurement of interepicondylar width and Baumann angle/humerus-elbow-wrist angle on plain radiograph; (2) compare differences between Baumann angle and humerus-elbow-wrist angle to measure the degree of cubitus angulation; and (3) identify the risk factors for cubitus varus.

## Materials & methods

A total of 342 children treated for lateral condylar fractures in one institute in 1998–2018 were enrolled. The data obtained from the patients’ medical records included age, sex, injury side, injury type (Song classification), treatment type, and X-rays. This retrospective chart review study involving human participants was in accordance with the ethical standards of the institutional and national research committee and with the 1964 Helsinki Declaration and its later amendments or comparable ethical standards. The Human Investigation Committee (IRB) of the institution approved this study. Baumann angle, humerus-elbow-wrist angle, and interepicondylar width were measured on standard elbow anteroposterior (AP) radiographs. Patients with unilateral lateral condylar fractures of the humerus with a follow-up period of over six months were included. Patients for whom X-rays were lacking, with bilateral involvement, or who were lost to follow-up were excluded. In pediatric elbow X-rays, positional problems due to rotation often occur. X-rays were assessed as to whether they were suitable for measurements as follows. (1) the elbow fully extended, in a supinated position with all aspects of the arm from the wrist to the humerus in the same plane; (2) centered at midpoint between the humeral epicondyles, superior to the distal third of the humerus and inferior to include one-third of the proximal radius and ulna. (3) patient’s arm rotated externally to ensure that the trochlea and capitellum are seen in profile. Patients with inappropriate X-rays were excluded from the analyses. A total of 150 children were excluded; thus, 192 children were retrospectively evaluated.

Of these 192 cases, 47 were treated non-surgically by immobilization, 110 by closed reduction and pinning, 31 by open reduction and internal fixation, and four by minimal invasive reduction and fixation with K-wires.

The demographic data were collected for all children. The fracture stages were categorized using the classification devised by Song et al [4]. Data on treatment modalities were obtained from their medical records. The age range at the time of trauma was 14 months to 11 years (mean age, 4.8 ± 2.0 years). There were 61 (31.1%) girls and 131 (68.9%) boys. The right elbow was affected in 75 (39.1%), while the left elbow was affected in 117 (60.9%). A total of 86 children were followed up until six months, 34 until one year, 28 until two years, and 44 for more than two years (Table 1). AP views of both elbows were obtained. Both elbow radiographs were routinely taken at the initial visit, six months and one year later, and every year thereafter if the child required further follow-up. Lateral bony overgrowth or angular deformity of the elbow occurs very often after lateral condyle fracture, slight changes all included. The deformity usually becomes apparent after six months from the injury. So after union of the fracture, bilateral AP x-rays annually is a simple but very necessary evaluation method to follow up complications of lateral condylar fracture.

**Table 1:** Demographic data

Characteristics	mean ± standard deviation (range) or Frequency (%)
Age (years)	4.8 ± 2.0
Sex	
Male	131 (68.9%)
Female	61 (31.1%)
Side	
Left	117 (60.9%)
Right	75 (39.1%)
Follow-up periods	
6 months	86 (44.8%)
1 year	34 (17.7%)
2 years	28 (14.6%)
> 2 years	44 (22.9%)
Treatment method	
Conservative	47 (24.5%)
Closed reduction pinning	110 (57.3%)
Open reduction pinning	31 (16.1%)
Other op. techniques	4 (2.1%)
Song stage	
1, 2	66 (34.4%)
3,4,5	126 (65.6%)
Baumann angle (°)	
Affected side	74.9 ± 6.8
Unaffected side	72.4 ± 5.2
Difference	2.5 ± 7.8
Humerus-elbow-wrist angle (°)	
Affected side	9.1 ± 4.4
Unaffected side	6.5 ± 5.5
Difference	2.9 ± 5.3
Interepicondylar width (mm)	
Affected side	46.1 ± 7.7
Unaffected side	42.6 ± 7.8
Difference	3.5 ± 3.0

A difference of more than 5° in varus angulation (Baumann angle or humerus-elbow-wrist angle) compared to the unaffected side was considered cubitus varus [12]. The interepicondylar width increase compared to the unaffected side was considered a result of lateral condylar overgrowth or a lateral bony spur.

**Fig. 2:**
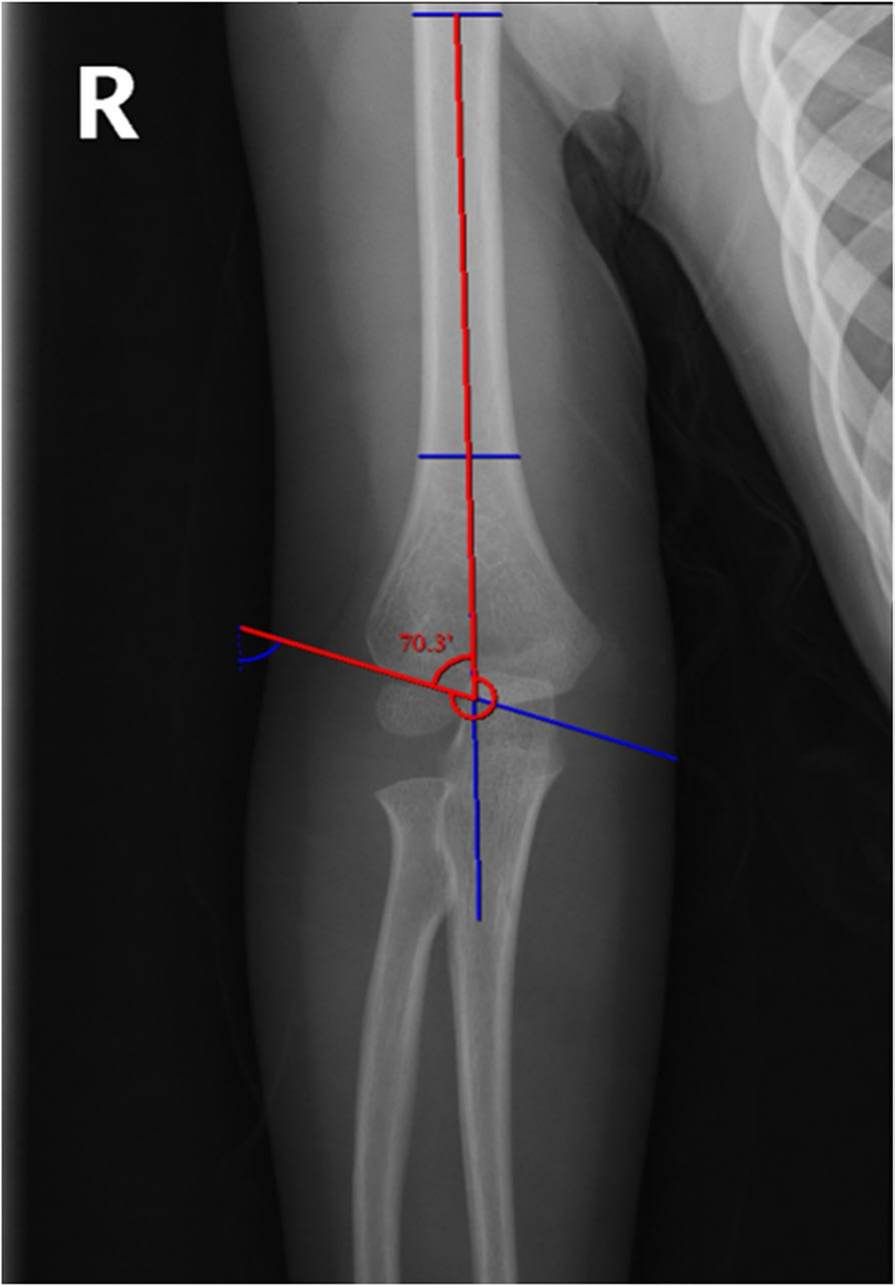
Radiograph illustrating the Baumann angle

**Fig. 3:**
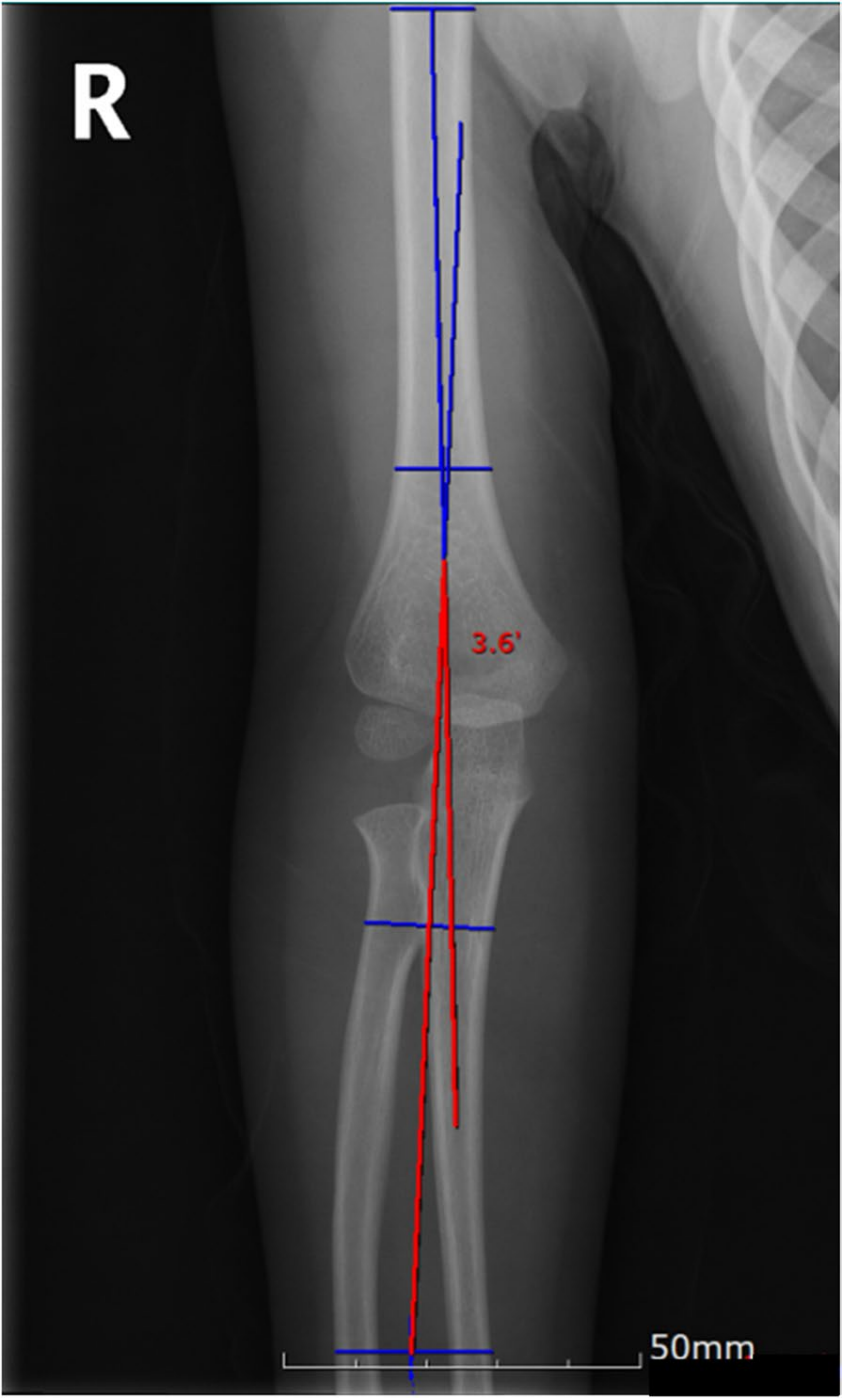
Radiograph illustrating the humerus-elbow-wrist angle

**Fig. 4:**
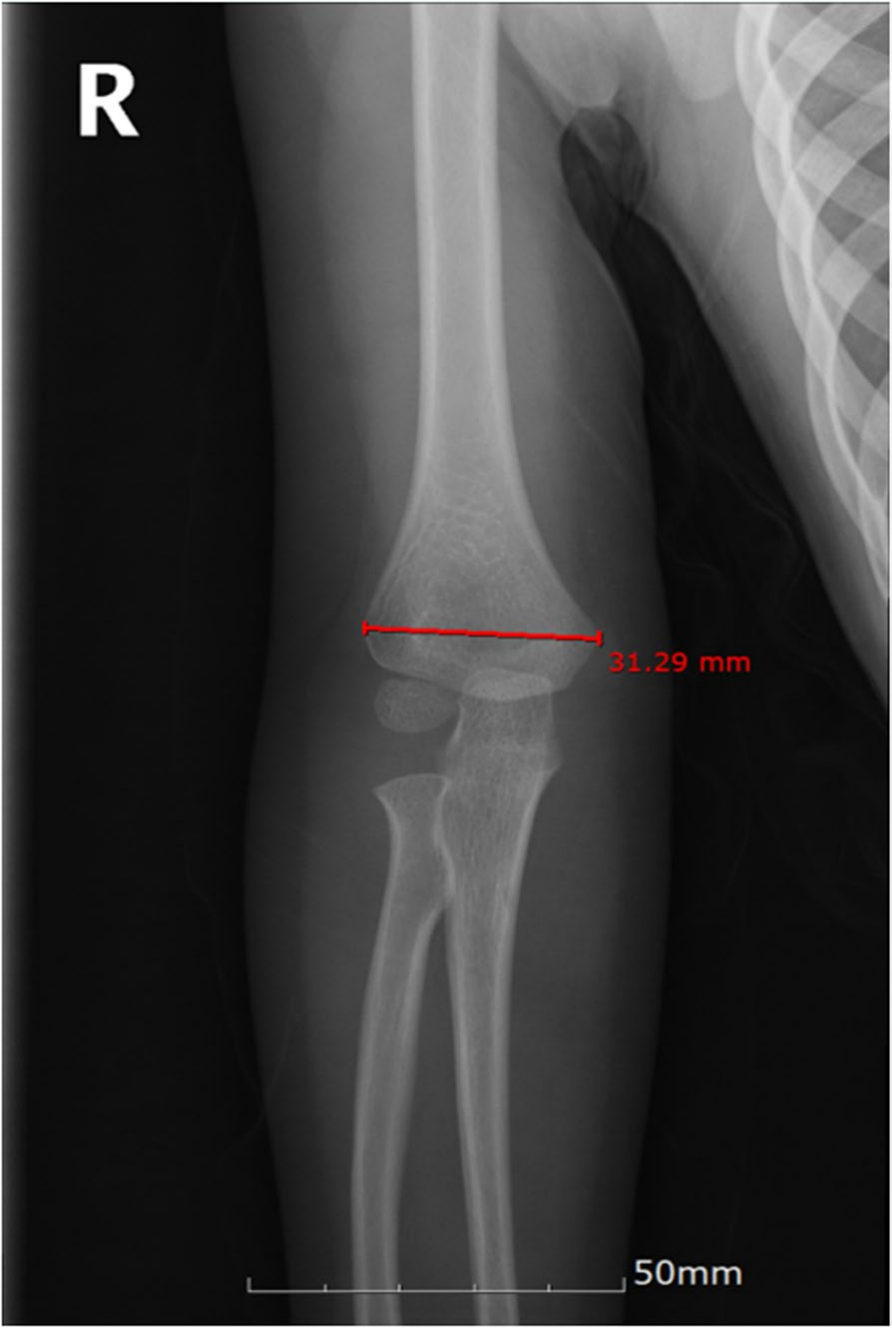
Radiograph illustrating the interepicondylar width

The Baumann angle is the angle between the longitudinal axis of the humerus shaft (the line passing through the midpoints of two transverse lines of the humerus shaft) and a line along the capitellar physis (Fig. 2). The humerus-elbow-wrist angle was measured as the angle between the longitudinal axis of the humeral shaft and a line passing through the midpoints of two transverse lines (one across the radial tuberosity and one distal) across the forearm (Fig. 3) [13]. The interepicondylar width was defined as the maximum distance between the medial and lateral epicondyles (Fig. 4) [14].

The Baumann angle, humerus-elbow-wrist angle, and interepicondylar width of both elbows were measured on the last follow up X-rays, by one pediatric orthopedic surgeon and one senior resident. The measurements were compared for interobserver reliability. The measurement by one pediatric orthopedic surgeon was utilized for other statistical analysis. Interobserver reliability was evaluated using the intraclass correlation coefficient (ICC). ICC less than 0.40 was interpreted as poor, between 0.40 and 0.59 as fair, between 0.60 and 0.74 as good, and between 0.75 and 1.00 as excellent [15]. The association between the Baumann angle and humerus-elbow wrist angle was evaluated using Pearson correlation coefficient. The association between the Baumann angle and interepicondylar width and association between humerus-elbow-wrist angle and interepicondylar width were also compared using Pearson correlation coefficient. The cut-off value of interepicondylar width for 5° varus angulation of the Baumann angle was predicted by ROC (Receiver Operating Characteristic) curve analysis. We also analyzed the risk factors that could predict the development of cubitus varus using logistic regression analysis. Statistical analyses were performed using IBM SPSS Statistics for Windows, Version 26.0 (IBM Corp., Armonk, N.Y., USA).

## Results

The interobserver reliability ICC for the Baumann angle, around 67%, humerus-elbow-wrist angle around 92%, and interepicondylar width around 97%. Good reliability was noted for the Baumann angle and excellent reliability for the humerus-elbow-wrist angle and interepicondylar width (Table 2).

**Table 2:** Interobserver reliability

	ICC (95% CI)	*P*-value
**BA unaffected side**	**0.66 (0.54–0.74)**	**< 0.01**
**BA affected side**	**0.68 (0.57–0.76)**	**< 0.01**
**HEWA unaffected side**	**0.90 (0.87–0.93)**	**< 0.01**
**HEWA affected side**	**0.93 (0.91–0.95)**	**< 0.01**
**IW unaffected side**	**0.97 (0.96–0.98)**	**< 0.01**
**IW affected side**	**0.98 (0.94–0.99)**	**< 0.01**

Values are intraclass correlation coefficient(ICC) with 95% confidence interval(CI)

*BA* Baumann angle, *HEWA* Humerus-elbow-wrist angle, *IW* Interepicondylar width

True cubitus varus was 32.8% measured by the Baumann angle and 29.2% measured by the humerus-elbow-wrist angle.

The interepicondylar width increase was 94.8%, reflecting lateral condylar overgrowth or a lateral bony spur. Pseudo-cubitus varus, which may appear as cubitus varus on gross inspection but without a true angular deformity of more than 5° than the unaffected side, 62% measured by Baumann angle and 65.6% measured by humerus-elbow-wrist angle. Overall, the incidence of pseudo-cubitus varus was 62–65.6%, while that of true cubitus varus was 29.2–32.8%.

**Fig. 5:**
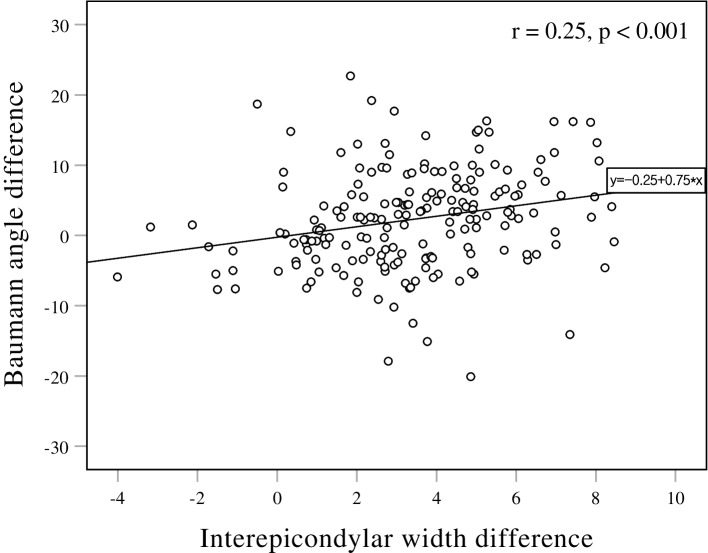
Correlation between Baumann angle and interepicondylar width

**Fig. 6:**
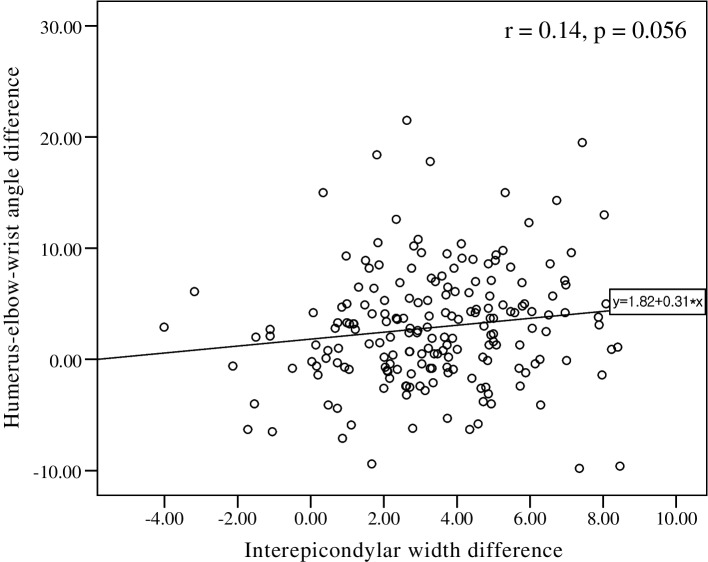
Correlation between humerus-elbow-wrist angle and interepicondylar width

**Fig. 7:**
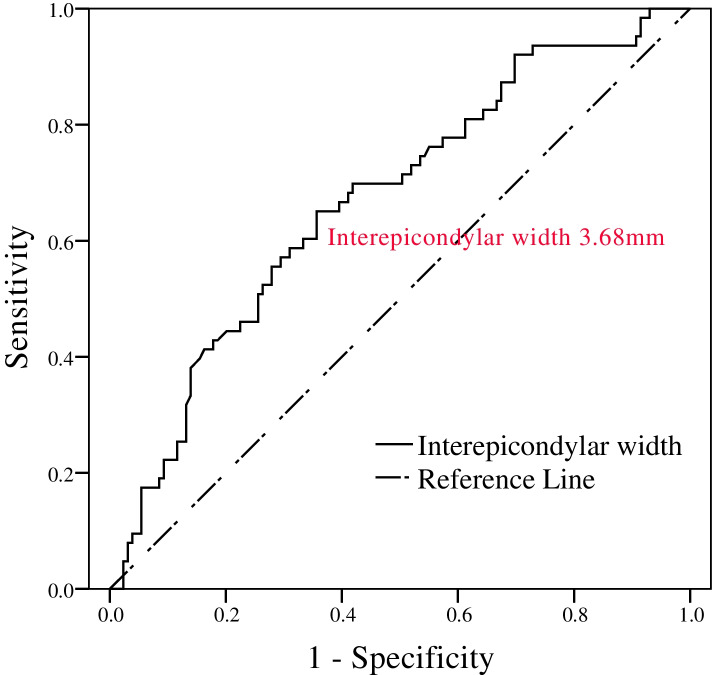
Receiver operating characteristic curve of usefulness of interepicondylar width difference for predicting a Baumann angle difference of more than 5°

The interepicondylar width had a statistically significant correlation with the Baumann angle (Fig. 5), but there was no correlation with the humerus-elbow-wrist angle (Fig. 6). The predicted cut-off value for 5° varus angulation on the Baumann angle was a 3.675 mm increase in interepicondylar width by ROC curve analysis (Fig. 7) (Table 3.). An interepicondylar width increase of over 3.7 mm compared to the unaffected side could predict a Baumann angle change of more than 5° in varus angulation.

**Table 3:** Baumann angle according to interepicondylar width increase in lateral condylar fracture

	Interepicondylar width > 3.7 mm	Interepicondylar width 3.7 mm or less	*p*-value
Baumann angle > 5°	41 (47.1)	22 (21.0)	< 0.001
Baumann angle 5° or less	46 (52.9)	83 (79.0)	

The risk of cubitus varus measured by Baumann angle in cases of stage 3, 4, and 5 fractures according to Song’s classification was 2.88 times higher than that of stage 1 and 2 fractures by multivariable logistic regression analysis after the adjustment for age, sex, direction, and treatment method (Table 4).

**Table 4:** Multivariable logistic regression analysis of risk factor for cubitus varus

	Odds ratio	(95% CI)	*p* value
**Age**	1.00	(0.99–1.02)	0.447
**Sex**	0.90	(0.42–1.93)	0.787
**Right/Left**	0.64	(0.32–1.27)	0.205
**Treatment method**			
** Conservative (reference)**	1.00		
** Closed reduction pinning**	1.59	(0.13–19.3)	0.714
** Open reduction pinning**	1.70	(0.16-18.0)	0.658
** Other op. techniques**	1.93	(0.16–23.4)	0.604
**Song stage**			
** 1,2 (reference)**	1.00		
** 3,4,5**	**2.88**	**(1.14–7.31)**	**0.025**

## Discussion

Cubitus varus and lateral overgrowth are reportedly the most common deformities after pediatric lateral condyle fractures (40% and 70%, respectively) [10, 11]. The X-ray findings may appear similar between these two complications. A lateral bony spur or lateral overgrowth could appear as cubitus varus, but without a true angular deformity, we defined it as pseudo-cubitus varus. Although they look alike, the clinical outcomes of true cubitus and pseudo-cubitus varus differ completely. Additional surgical procedures, including corrective osteotomy, might be necessary when cubitus varus becomes a problem [16]. On the other hand, pseudo-cubitus varus usually does not require treatment [14]. Therefore, differentiating pseudo-cubitus varus from cubitus varus may be essential.

Previous studies reported the presence of a lateral spur in up to 70% of cases after pediatric lateral condyle fractures [10]. In a recent study, lateral overgrowth was measured as the maximum interepicondylar width on the initial and final AP radiographs and defined as an increase in interepicondylar width of 100–110%, moderate spurring as 110–120% increase, and severe as > 120% [14]. In this study, lateral condylar overgrowth or a lateral bony spur was observed in 94.8% of patients. Even a slight increase in interepicondylar width compared to the unaffected side was considered lateral condylar overgrowth in this study, which resulted in a higher prevalence than that reported in previous studies.

In previous cubitus varus studies, various methods including Baumann angle and carrying angle, were used to measure the cubitus angle [10, 17]. Recently, the humerus-elbow-wrist angle has emerged as a reliable measurement method since good inter- and intraobserver reliabilities were correlated with other measurement methods [13].

In previous studies, the prevalence of cubitus varus after lateral condyle fractures in children was 40% [11]. In this study, the prevalence of cubitus varus after lateral condyle fractures in children was 30%. The definition of cubitus varus was a difference of more than a 5° angle in this study [12], which might have been the cause for this difference.

In this study, an increase in interepicondylar width could measure and define lateral overgrowth of bony spur and predict true cubitus varus. Lateral condylar overgrowth or lateral bony spur occurred in 94.8% of patients. Pseudo-cubitus varus occurred in 62% as measured by Baumann angle and 65.6% as measured by humerus-elbow-wrist angle. Cubitus varus occurred in 29.2–32.8%. Statically, the Baumann angle had a valid correlation with interepicondylar width. The predicted cut-off value for the 5° difference in varus angulation on the Baumann angle was a 3.675 mm increase in the interepicondylar width. An interepicondylar width increase of greater than 3.7 mm compared to the unaffected side could predict a Baumann angle change of more than 5° in varus angulation (sensitivity 65.1%, specificity 64.3%, positive predictive value [PPV] 47.1%, negative predictive value [NPV] 79.0%). Larger interepicondylar width may have resulted from a higher stage of Song’s classification which is related with a higher risk of cubitus varus. The Baumann angle can show inter- or intraobserver differences in the determination of capitellar physis, especially after physeal closure. The humerus-elbow-wrist angle can differ with the locations of the two transverse lines across the forearm, which was defined as one across the radial tuberosity and one distal. Since interepicondylar width measurements are much more convenient to make than the Baumann angle or humerus-elbow-wrist angle, the possibility of inter- or intraobserver differences would be decreased compared to the other two measurement methods.

This study was the first to compare cubitus varus according to the fracture stage classification of Song et al. [4]. Previous study compared the Jakob classification, and lateral bony overgrowth did not differ between subtypes [10]. This study found a statistically significant correlation between initial fracture severity according to Song stage and the incidence of cubitus varus. Song stage 3, 4, and 5 fractures initially had a 2.88-times higher risk of cubitus varus than stage 1 and 2 fractures. However, although operation was indicated for Song stage 3, 4, and 5 lateral condyle fractures, operation was not a significant risk factor for cubitus varus. Despite treatment modality, the initial lateral condyle fracture severity affected the risk of cubitus varus.

This study has several limitations. First, open anatomical reduction compared to closed reduction would have influenced the results [18]. More complications are trending in less anatomical reductions, such as angular deformity or lateral bony growth. But open reduction is usually necessary for more displaced (or for higher stage) fractures. The more severe the damage from the injury, the greater the possibility of growth disturbance. Comparing one factor regardless of other factors is difficult, because they are all closely related. Second, similar to many other studies using X-rays, there are radiographic limitations. Position, alignment, and rotations of the patient, magnification of the image, and image quality differ among films. Therefore, to make an accurate comparison, placement of a calibration marker on the patient for reference is required. Finally, we also wanted to determine the point of maximal overgrowth or varus angulation, and when the lateral bony spur remodeled. However, an insufficient number of patients completed long-term follow-up. Follow-up intervals were not equivalent between patients. In future studies, it would be better to complete longer follow-up periods with regular intervals to determine when the remodeling process occurs most often and if the varus deformity or lateral spur spontaneously decreases over a certain period.

This study used a large database of pediatric patients to determine the relationship between true cubitus varus and pseudo-cubitus varus by measuring interepicondylar width, Baumann angle, and humerus-elbow-wrist angle. In contrast to previous studies, Song classification was applied to perform risk evaluations of true cubitus varus.

## Conclusion

Pseudo-cubitus varus showed a higher prevalence than true cubitus varus. A 3.7 mm increase in interepicondylar width compared to the unaffected side could simply predict true cubitus varus. The risk of cubitus varus was increased for Song stages 3, 4, and 5 fractures.

## Data Availability

The datasets generated during and/or analyzed during the current study are available from the corresponding author on reasonable request.

## Abbreviations

ROC: Receiver Operating Characteristic
IRB: Institutional Review Board
AP: Anteroposterior
ICC: Intraclass Correlation Coefficient
PPV: Positive Predictive Value
NPV: Negative Predictive Value
